# 46,XX Testicular Disorders of Sex Development With *DMD* Gene Mutation: First Case Report Identified Prenatally by Integrated Analyses in China

**DOI:** 10.3389/fgene.2019.01350

**Published:** 2020-02-19

**Authors:** Jianlian Deng, Haoqing Zhang, Caiyun Li, Hui Huang, Saijun Liu, Huanming Yang, Kaili Xie, Qiong Wang, Dongzhu Lei, Jing Wu

**Affiliations:** ^1^School of Future Technology, University of Chinese Academy of Sciences, Beijing, China; ^2^BGI-Shenzhen, Shenzhen, China; ^3^Center of Prenatal Diagnosis, Chenzhou No.1 People’s Hospital, Hunan, China; ^4^BGI Genomics, BGI-Shenzhen, Shenzhen, China; ^5^James D. Watson Institute of Genome Sciences, Hangzhou, China; ^6^Division of Obstetrics，Zhuzhou Central Hospital, Hunan, China; ^7^Genetic Eugenics Division, The Maternal and Child Health Hospital of Changde City, Hunan, China

**Keywords:** integrated analyses, 46,XX testicular DSD, Duchenne muscular dystrophy, prenatal, genetic counseling

## Abstract

The present study describes the first prenatally diagnosed 46,XX testicular disorders of sex development (46,XX testicular DSD) case with *DMD* gene mutation by integrated analyses in a Chinese pedigree. Chromosome karyotype G-banding analysis of the proband showed a 46,XX karyotype, but B-ultrasound analysis demonstrated the existence of scrotum, testis and penis which inferred a male sexual differentiation. Aneuploidy and copy number variation (CNV) detection by low-coverage single-end whole genome sequencing (WGS) revealed a *de novo SRY* (sex-determining region Y) gene positive fragment of 224.34 kb length (chrY:2,649,472-2,873,810) which explained the gonadal/genital-chromosomal inconsistency in the proband. Additionally, targeted-region-capture-based *DMD* gene sequencing and Sanger verification confirmed a widely reported pathogenic heterozygous nonsense mutation (NM_004006, c.9100C>T, p.Arg3034Ter) in the dystrophin-coding gene named *DMD*. This study emphasizes that integrated analyses of the imaging results, cytogenetics, and molecular features can play an important role in prenatal diagnosis. It requires the combination of more detection techniques with higher resolution than karyotyping to determine the genetic and biological sex of fetuses in prenatal diagnosis. To conclusively determine both the biological and genetic sex of the fetus at the time of prenatal diagnosis particularly in cases that involve X-linked conditions is of vital importance, which would crucially influence the decision-making regarding abortions. This study will help in prenatal diagnosis of DMD in future, also providing a new perspective that enables the genetic diagnosis of sex reversal in pregnancy. Moreover, genetic counseling/analysis for early diagnosis and pre-symptom interventions are warranted.

## Background

Abnormalities in chromosomes or genetic materials can lead to birth defects. The term 46,XX testicular disorders of sex development (46,XX testicular DSD), previously termed as male sex reversal, was introduced by the “Chicago Consensus” in 2006, for individuals who manifest inconsistency of chromosomal, gonadal, or anatomical sex with a male phenotype and a female karyotype (Hughes et al., 2006) and was firstly described by de la Chapelle et al. in 1964 (De la Chapelle et al., 1964). The occurrence rate of 46,XX testicular DSD ranges from 1/20,000 to 1/100,000 (de la Chapelle, 1981; SS, 1994; Berglund et al., 2017; Wang et al., 2017), with considerable geographic variations. Approximately 80% of individuals affected by 46,XX testicular DSD show typical male phenotype at birth, and present with infertility or delayed puberty. Most are diagnosed during adolescent stage (Ergun-Longmire B et al., 2005). In the Y chromosome, the *SRY* gene is heavily involved in encoding a testis determining factor (TDF) that initiates male sex determination and controls testis differentiation. Abnormal expression of *SRY* gene may influence the testicular differentiation, leading to ambiguous male phenotype (Sinclair AH et al., 1990). Deletions of regions termed as azoospermia factors in long arm of Y chromosome (i.e, AZFa, AZFb, AZFc, and AZFd) were determined to be associated with spermatogenesis and leads to testiculopathy. Severe testiculopathy, in turn, results in azoospermia or severe oligozoospermia (Raicu et al., 2003). About 90% of 46,XX testicular DSD patients with a male phenotype are *SRY* positive (Rizvi, 2008; Ahmet Anık, 2013; Gunes et al., 2013; Wang et al., 2009), although published *SRY*-negative studies mostly supported the role of *SOX* family proteins in male sexual development with a proportion of 10 to 15% (Huang et al., 1999; Lim et al., 2000; Seeherunvong et al., 2004; Grinspon and Rey, 2016). Recently, duplications of testicular *SOX9* enhancers have been reported to result in SOX9 overexpression and act as a significant cause of 46,XX DSDs (Croft et al., 2018). Detection of *SRY* gene and azoospermia factors is essential for the diagnosis of 46,XX testicular DSD.

Duchenne muscular dystrophy (DMD, OMIM #310200) which transmits in an X-linked recessive pattern is the most severe and rapidly progressive type of dystrophinopathies. There is significantly massive increase of creatine kinase levels in the blood, electromyography usually shows myopathic changes. Individuals affected by DMD experience motor defects resulting from progressive loss of muscle function. DMD is caused by mutations in the dystrophin-coding gene named *DMD* and primarily affects boys while occasionally affects girls. Symptom onset is usually between ages 3 and 5, with an incidence of 1/3,500 in newborn males (Matsuo, 1995; Prior and Bridgeman, 2005; Centers for Disease, C. and Prevention, 2009). In clinical genetic counseling of DMD, prenatal diagnosis is recommended for pregnant female carriers with a DMD-positive childbearing history to make an informed decision and to understand their chances of bearing a child with detectable abnormality to prevent recurrence in at-risk families (Than and Papp, 2017).

There have been rarely reported cases who are affected simultaneously by both 46,XX testicular DSD and DMD; and specifically, 46,XX testicular DSD cases are rarely prenatally identified in individuals owing to its mostly normal male appearance before puberty, despite that there has been a 46,XY DSD case report by prenatal diagnosis who showed positive family history (Mazza et al., 2003).

Recent implementation of genetic or chromosomal diagnostic techniques for birth defects include: karyotype analysis, fluorescence *in situ* hybridization (FISH), array comparative genomic hybridization (array CGH), and next generation sequencing (NGS) for detection of genetic abnormalities. It is difficult to detect chromosomal abnormalities below 5 Mb in conventional karyotyping, FISH technology with higher sensitivity and specificity can only be used for a specifically targeted diagnosis to exclude certain diseases, while array CGH can only detect unbalanced chromosomal abnormalities and the result could be largely affected by the probe density. However, complete gene sequencing by target-captured NGS has been focusing on small point mutations, as well as mechanisms related to the etiology of diseases. Research studies show that NGS-based low-coverage whole genome sequencing (WGS) could rapidly detect fetal aneuploidies and microdeletions/microduplications, being an alternative noninvasive option to current detection methods for disease diagnosis (Dan et al., 2012; Baxter and Vilain, 2013; Chen et al., 2013; Xie et al., 2013).

The present study aimed to prenatally identify a Chinese patient with 46,XX testicular DSD and *DMD* gene mutation by integrated analyses.

## Case Presentation

The study was carried out in accordance with the tenets of the Declaration of Helsinki and approved by the institutional review boards of BGI and Chenzhou No.1 People’s Hospital. Written consent was obtained from each participant (or legal guardian if incapacitated).

**Figure 1:**
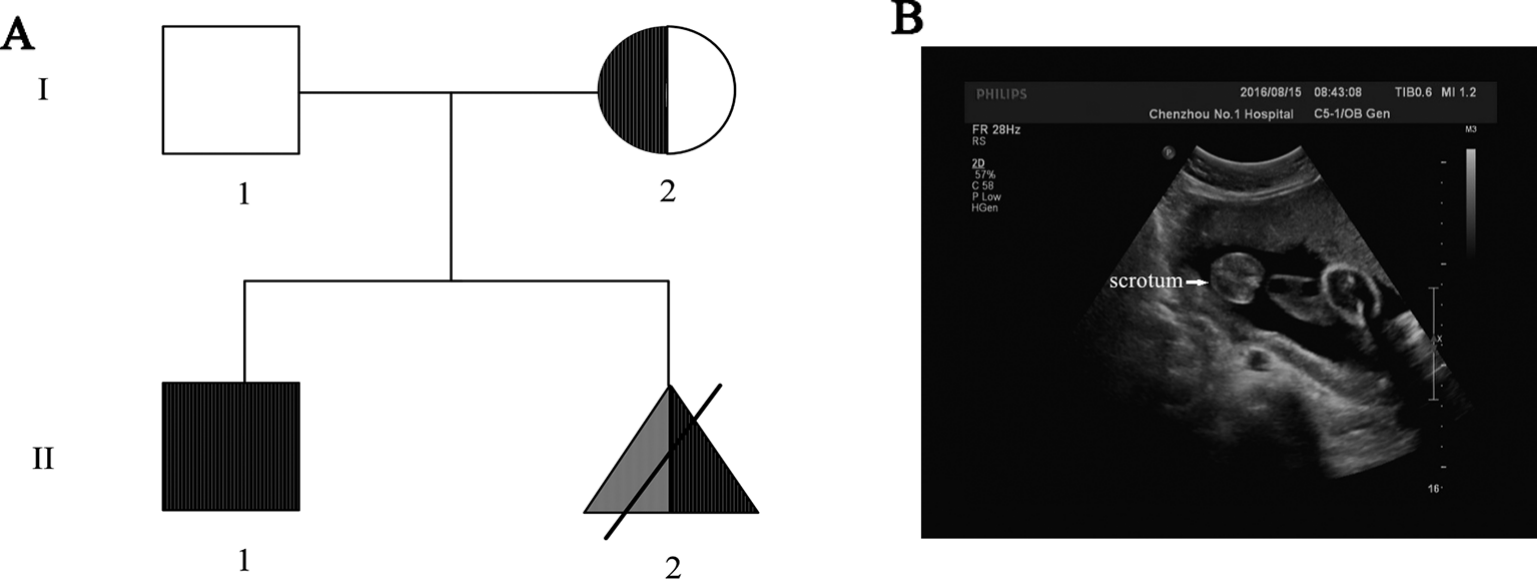
**(A)** Pedigree of the Chinese family. The fully filled symbol in black indicates those affected by Duchenne muscular dystrophy (DMD), and the open symbol indicates those unaffected; the filled semicircle represents asymptomatic carriers. Squares represent male, circles represent female, specifically, triangle represents the labor-inducted fetus—the proband with 46,XX testicular DSD (filled in grey) and *DMD* gene mutation (filled in black). **(B)** Ultrasound result of the fetus (27th week of gestation) showed the existence of scrotum, indicating male gonadal differentiation.

Four members of a Han Chinese nonconsanguineous family were recruited (**Figure 1A**). The proband was an unborn baby (27th week of gestation) who was the junior of two siblings from the family which showed no family history of 46,XX testicular DSD. The 6-year-old brother of the proband was clinically and genetically diagnosed with DMD: he was found to be walking wrestling in his third year, physical examination showed gastrocnemius muscle hypertrophy, while limb muscle tension and strength were both tolerable; test results of Kernig/Brudzinski/Babinski sign were negative and Gowers sign was positive; electromyography (EMG) results suggested myogenic damage; the concentrations of creatine kinase and creatine kinase isoenzyme were 16347 u/l and 373 u/l, respectively, while the concentration of myoglobin was 544.7 ng/ml, which were strong evidences for DMD diagnosis. The brother was identified carrying a widely known pathogenic mutation (NM_004006, c.9100C>T, p.Arg3034Ter) in *DMD* gene by targeted gene sequencing. Parents of the proband were both phenotypically normal.

Due to the positive family history of DMD and the existence of scrotum in ultrasound result indicating male gonadal differentiation of the proband (**Figure 1B**), genetic counseling and prenatal diagnosis were recommended to the proband.

## Materials and Methods

The clinical manifestations, molecular features, and genotype-phenotype correlations of the proband were studied.

### DNA Samples Preparation

Cord blood and labor-induced tissue samples of the proband, peripheral blood samples of the older brother and mother of the proband were taken at Chenzhou No.1 People’s Hospital in 2016. Genomic DNA was isolated using QIAamp DNA BloodMiNi kit (Qiagen, Hilden, Germany) for blood samples and QIAamp DNA FFPE Tissue Kit (QIAGEN, Frankfurt, Germany) for tissue, respectively.

### Cytogenetics Analysis by G-Banding Karyotyping

Standard G-banding cytogenetics analysis was performed with chromosomes isolated from leukocytes in 5 ml umbilical cord blood obtained from ultrasound-guided umbilical vein puncture for prenatal diagnosis. The G-banding steps are listed below: cell culture was performed with medium produced by Bosheng Company (Hangzhou,China) according to the specific protocols; the cultured cells were treated in the following orders: (1) Colcemid treated (0.05 mol.L^-1^ Colcemid, 40 μl, 4 h, 37°C) and centrifuged (978 xg, 10 min); (2) hypo-osmotically treated (0.075 mol.L^-1^ KCL hypotonic solution, 8 ml, 35 min, 37°C); (3) prefixed (1.5 ml fixative by 3:1 ratio of methanol:glacial acetic acid, 10 min, 37°C) and centrifuged (978 xg, 10 min); (4) fixed twice (6 ml fixative by 3:1 ratio of methanol:glacial acetic acid, 10 min 37°C). After these, cells were prepared for cytogenetics analysis. Slides for G-Banding were prepared after the cell suspension was dried upon an ice slide in a 75°C oven for 3 h. The slides were digested with 1.5 ml 0.025% trypsin, then stained with 6% Giemsa for 10 min (37°C, pH 6.2). Karyotype analysis was performed and the chromosomal anomalies were reported according to the International Human Cytogenetics Nomenclature System (ISCN2016) standard. All the operations were carried out by two trained technicians independently.

### Prenatal Diagnosis by Targeted-Region-Capture-Based *DMD* Gene Sequencing and Sanger Verification

Total DNA from cord blood was extracted using QIAamp DNA BloodMiNi kit (Qiagen, Hilden, Germany) and sonicated into 200–250 bp fragments by an ultrasonoscope (Covaris S2; Covaris,Inc., Woburn, MA, USA). The fragmented products were progressed with adaptor ligation, which was followed by amplification with a gradient PCR program, containing a Pfx DNA polymerase (Invitrogen Life Technologies, Calsbad, CA, USA) with high-fidelity. PCR reactions were conducted in the Veriti PCR system (Applied Biosystems Life Technologies, Foster City, CA, USA) with reference to the Tm during primer designation with Primer 6. The first phase was 5 min at 95˚C, the second phase was 30 s at 95˚C, 30 s at 68˚C, and 30 s at 72˚C for 10 cycles, the third phase was 30 s at 94˚C, 30 s at 58˚C, and 30 s at 72˚C for 35 cycles, followed by the fourth phase with 5 min at 72˚C, and last by 4˚C. The custom-designed capture array targeted all exons and the flanking 20 bp noncoding sequence of *DMD* gene, 100-bp pair-end sequencing was performed on Illumina HiSeq2500 Analyzers after which image analysis and base calling were performed with Illumina Pipeline software (version 1.3.4) with the human reference genome (hg19, NCBI build 37). Candidate SNPs were filtered with the following criterions: SNP supporting reads ≥20, SNP frequency ≤5% in any of the following three databases (dbSNP, Hapmap, 1000 Genomes Project). Sanger sequencing was applied to validate the candidate mutations on ABI PRISM 3730 sequencer (Applied Biosystems, Foster City, CA, USA) and analyzed by DNASTAR SeqMan (DNASTAR, Madison, Wisconsin, USA).

### Genetic Abnormalities Detection by NGS-Based Low-Coverage Single-End WGS

Genomic DNA from the proband’s labor-aborted tissue and cord blood were detected for subsequent genetic abnormalities using NGS-based low-coverage single-end WGS on BGISEQ-500 platform and CNVs were analyzed as we previously reported (Dan et al., 2012; Dong et al., 2014; Li et al., 2014).

### Cellular Histopathological Analysis

Postmortem study after labor abortion was carried out for cellular histological examination. Fresh specimens were processed to formalin fixed, paraffin embedded (FFPE) tissue samples and stained with hematoxylin and eosin (i.e., H and E staining) with reference to the widely used method^1^.

### AZF Deletion Analysis by Multiple PCR

200~500 bp DNA sequence fragments termed sequence-tagged sites (STSs) could be considered as markers for genetically physical mapping and used in genetic screening (Olson et al., 1989). We performed multiple PCR of 15 STSs to detect microdeletions in the spermatogenesis and testiculopathy-associated regions (i.e., AZFa, AZFb, AZFc, and AZFd in the AZF region of proband’s *SRY* gene) to characterize the future fertility status and possible genotype-phenotype correlations of the proband. Totally, three sites in AZFa, six sites in AZFb, four sites in AZFc, and two sites in AZFd were studied.

## Results

### Prenatal Diagnosis and Pedigree Verification of DMD

**Figure 2:**
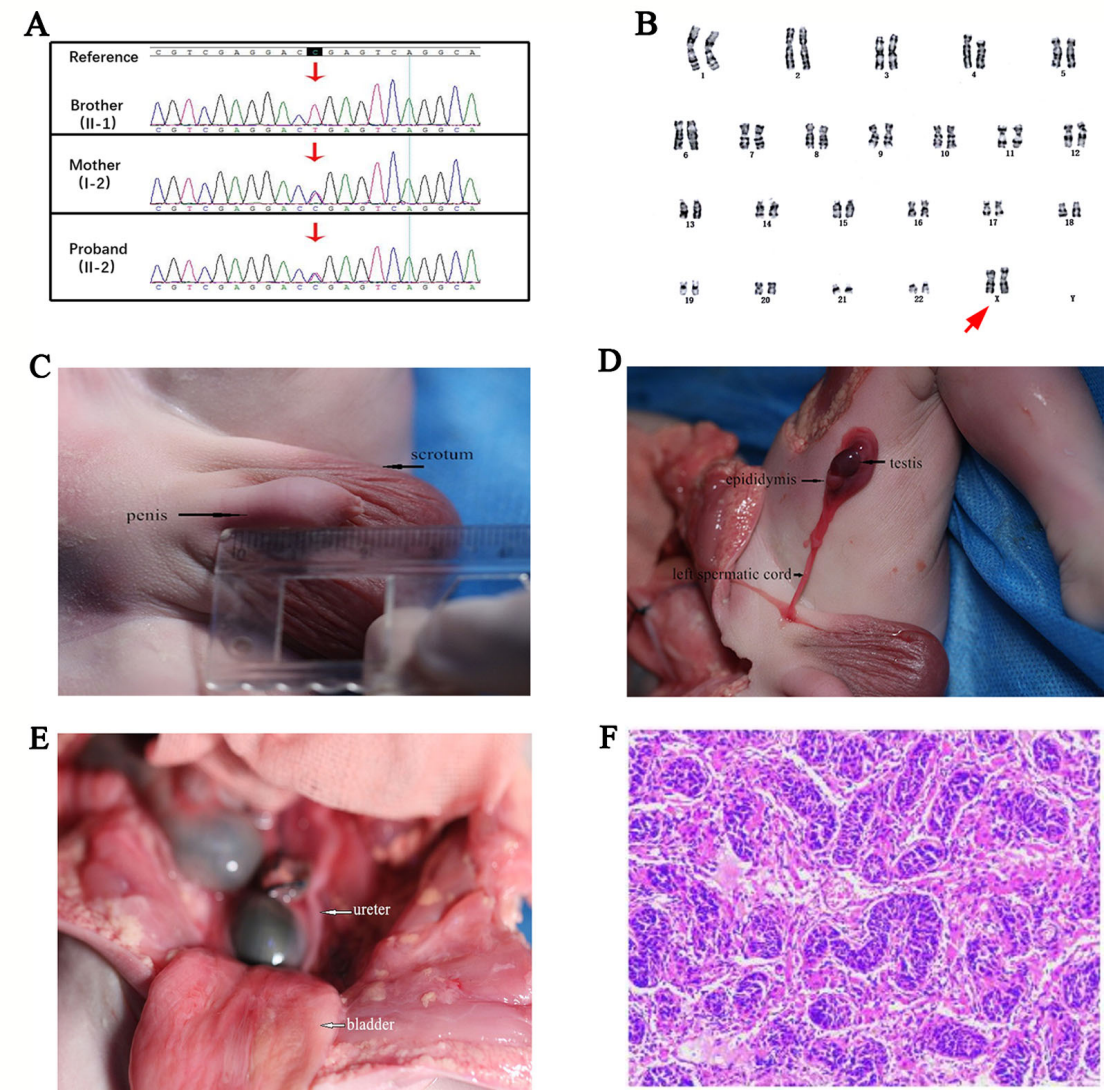
**(A)** Pedigree verification results of the c.9100C>T mutation in Duchenne muscular dystrophy (*DMD)* gene of the older brother (hemizygous), the asymptomatic mother (heterozygous), and the proband (heterozygous) by Sanger sequencing. **(B)** Standard cytogenetics by G-banding karyotyping of the fetus’s lymphocytes revealed a 46,XX karyotype. The arrow referred to the proband’s XX chromosomes. **(C)** Morphological examination showed the appearance of scrotum and penis, and the penis was with a length of about 19 mm, indicating a gonadal male sexual differentiation. **(D)** Autopsy operation revealed the presence of testis, epididymis, and left spermatic cord. **(E)** The ureter and bladder of the fetus were shown. **(F)** Histopathological result of testis biopsies showed the appearance of an embryonic testis with no fibrosis and hyaline degeneration of the tissue (Light microscopy stained with HE. Original magnification x 40).

Targeted *DMD* gene testing of the fetus for prenatal diagnosis revealed that the proband also carried the widely reported pathogenic heterozygous truncated mutation (c.9100C>T, p.Arg3034Ter) as his older brother did. Sanger sequencing for pedigree analysis confirmed that their asymptomatic mother also carried this mutation (**Figure 2A**). The proband and his DMD-affected brother both inherited this mutation from their asymptomatic mother.

### Integrating Analysis Results Confirmed a Diagnosis of 46,XX Testicular DSD

**Figure 3:**
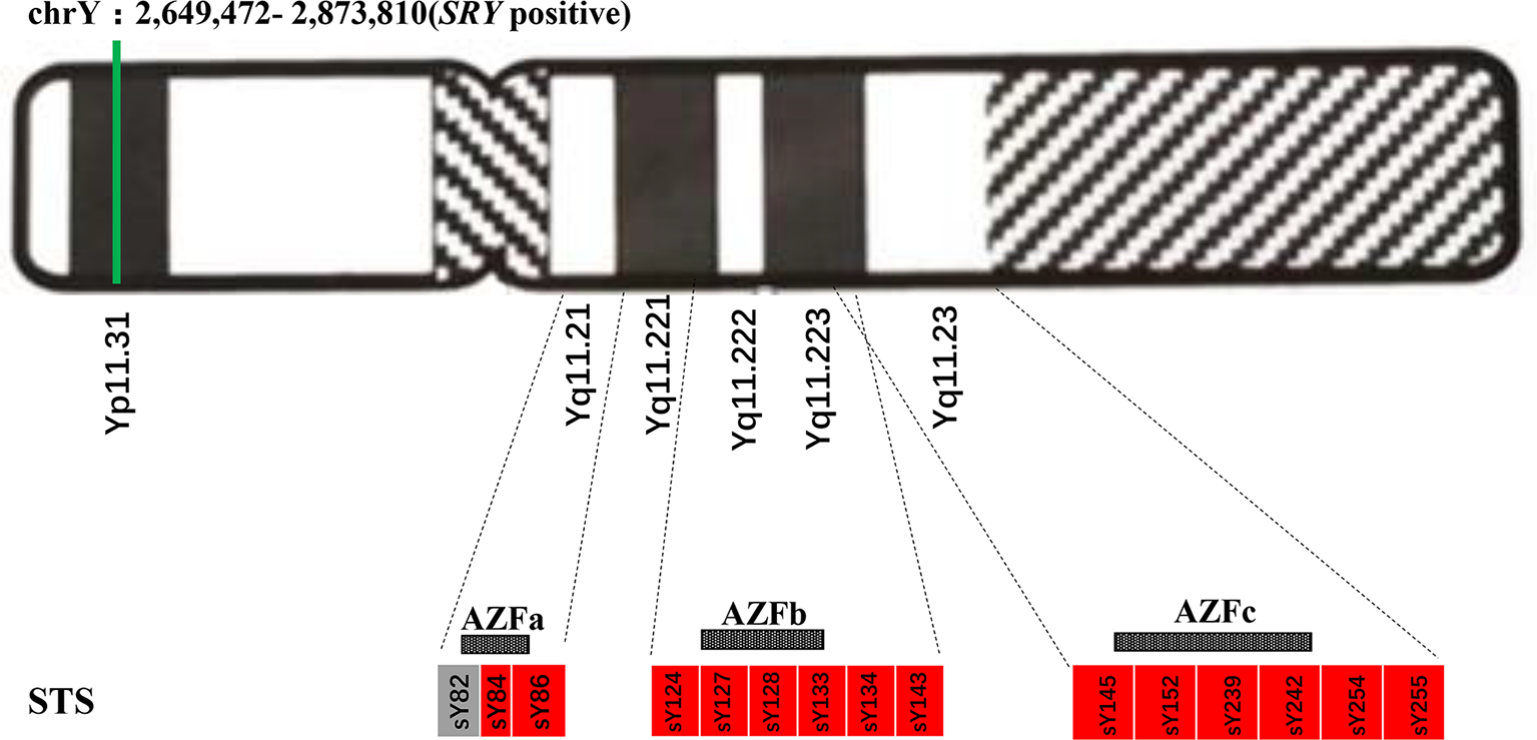
Azoospermia factor (AZF) deletions detected by multiple polymerase chain reaction (PCR) of sequence-tagged sites (STSs) in the proband. The green line represents the existence of the sex-determining region Y (*SRY*)-positive fragment of Y chromosome. Boxes represent AZF regions: grey-filled indicates the existence while red-filled indicates deletion.

Standard G-banding karyotype analysis of the proband’s lymphocytes showed a 46,XX karyotype (**Figure 2B**), unexpectedly, in inconsistency with the clinical indications by ultrasound which displayed the existence of scrotum (**Figure 1B**), raising a suspect diagnosis of 46,XX testicular DSD. To confirm this, aneuploidy and CNV detection was carried out by NGS-based low coverage single-end WGS, which identified a *de novo SRY* positive gain fragment of 224.34 kb length (chrY:2,649,472-2,873,810) in the proband. The variations identified in this research were all shown in **Table 1**. We then evaluated the clinical features of the proband after labor-abortion, which depicted a male histopathological phenotype (**Figures 2C–F**). Deletion analysis of regions termed as azoospermia factors (i.e, AZFa, AZFb, AZFc, and AZFd) in long arm of Y chromosome by analyses of 15 sequence-tagged sites in the AZF region by multiple PCR showed deletions in several AZF subregions (**Figure 3**).

**Table 1:** Variations identified by next-generation sequencing (NGS)-based low-coverage whole-genome sequencing in the proband, including a pathogenic and three VUS (variant of unknown significance) or likely nonpathogenic ones.

Variations	Fragment size	Genes enrolled in the regions	Clinical significance
**46, XX, +Yp11.31. seq[GRCh37/hg19](2,649,472-2,873,810)×1**	**224.34 kb**	***SRY***	**pathogenic**
46,XX,dup(6q27).seq[GRCh37/hg19] (169,990,779-170,201,296)×3	210.52 kb	*WDR27;PHF10;TCTE3; LINC00574;C6orf70; C6orf120;LINC00242*.	VUS/likely nonpathogenic
46,XX,dup(9p21.1).seq[GRCh37/hg19] (28,566,974-28,667,973)×3	101.00 kb	*LINGO2*	VUS/likely nonpathogenic
46,XX,dup(14q12).seq[GRCh37/hg19] (27,799,966-28,452,468)×3	652.50 kb	*LOC100505967*	VUS/likely nonpathogenic

The bolded texts referred the pathogenic SRY gene positive fragment which explained the gonadal/genital-chromosomal inconsistency in the proband.

## Discussion

This is the first 46,XX testicular DSD case with *DMD* gene mutation who is prenatally diagnosed by integrated analysis. In clinical genetic counseling, prenatal diagnosis is recommended for pregnant female carriers with a family history of DMD to prevent recurrence in at-risk families. Some researchers even believed that prenatal diagnosis was also suggested for the proband’s mother without the causative mutation in their blood considering maternal germline mosaicism (Wang et al., 2017). In this study, the mother (I-2) had raised a DMD-affected boy, so we carried out targeted *DMD* gene testing and Sanger sequencing in present family for prenatal diagnosis, which identified that the fetus (II-2) also carried the widely reported pathogenic mutation (c.9100C>T, p.Arg3034Ter) (Dent et al., 2005; Flanigan et al., 2009; Magri et al., 2011) in a heterozygous state. At the same time, we also identified in the proband a *de novo* sex-determining region Y gene (*SRY*) positive gain fragment of 224.34 kb length (chrY:2,649,472-2,873,810) by NGS. The presence of a Y DNA fragment containing *SRY* gene, primarily due to abnormal Y/X chromosomal exchange during paternal meiosis, was reported as the major cause of 46,XX testicular DSD (Rizvi, 2008; Wang et al., 2009; Ahmet Anık, 2013; Gunes et al., 2013; Wu, Li et al., 2014). These studies showed that those patients commonly had normal male external genitalia, small testis was frequently present and they always had azoospermia. The *SRY* gene in this fragment could initiate the testicular differentiation and the CNV was thus defined as pathogenic according to ACMG (American College of Medical Genetics and Genomics) guidelines (Kearney et al., 2011; South et al., 2013; Richards et al., 2015) which explained the gonadal/genital-chromosomal inconsistency. We mapped the other three microduplications identified in this research (**Table 1**) to the DGV database (Database of Genomic Variants^2^), which is a curated catalogue of human genomic structural variations (MacDonald et al., 2014). The results showed that all these three microduplications seemed probably to be nonpathogenic: the 210.52 kb duplication in 6q27 (169,990,779-170,201,296) involves the periventricular nodular heterotopia 6 (OMIM #615544)-associated *ERMARD* gene, but this region is all included by DGV; the 101 kb duplication in 9p21.1 (28,566,974-28,667,973) and 652.5 kb duplication (27,799,966-28,452,468) in 14q12 both do not contain any known disease-causing genes and there has been no publication for their pathogenicity.

Most heterozygous female carriers of *DMD* pathogenic mutations are asymptomatic. Considering the confirmed 46,XX karyotype by cytogenetic karyotyping, the proband here was most likely to be just a asymptomatic carrier as his mother. But it has also been reported that about 2.5% to 7.8% of these carriers are manifesting carriers who develop symptoms ranging from mild muscle weakness to a rapidly progressive DMD-like muscular dystrophy with an onset age varying from 2 to 47 years old (Soltanzadeh et al., 2010). The symptoms of a manifesting female carrier may due to X-inactivation of the normal dystrophin (*DMD*) gene (Boyd et al., 1986). In females, the random X inactivation process that happens in somatic cells early during embryonic development regardless of the parental origin, arouses cellular mosaicism in females with either paternally or maternally derived X-chromosome being inactivated (Brown and Robinson, 2000; BR, 2007). Payam Soltanzadeh (Soltanzadeh et al., 2010) reported a female manifesting carrier of the p.Arg3034Ter mutation who presented mild Becker’s muscular dystrophy (BMD)-like syndromes including weakness, myalgia/cramping with an onset age at 40; she was walking unaided until 47 years old. In another study by Forbes, a female manifesting carrier of the same mutation with an onset age of 37 showed substantial muscle deterioration and lipid infiltration heterogeneously detected by magnetic resonance imaging (MRI) and spectroscopy (MRS) (Forbes et al., 2012). It has been reported that symptomatic BMD carriers show a skewed XCI pattern with a preferential inactivation of the wild-type X chromosome (Viggiano et al., 2017). According to Viggiano’s theory, these two symptomatic female carriers would have larger proportion of cells that have wild-type chromosome inactivated than those have Arg3034Ter-mutated chromosome inactivated.

Furthermore, structural abnormalities including deletions and duplications of the X chromosome could cause occurrence of marked skewing of XCI (MF, 2002; Sharp, Kusz et al., 2004). In light of 46,XX testicular DSD, about 90% patients are *SRY* positive, usually translocated to the short arm of the X or autosomal chromosome, leading to a male phenotype (McElreavey and Cortes, 2001). However, variation of the phenotypic features of the *SRY* positive 46,XX males were observed owing to other factors including the length of the Y chromosomal material translocated on the X chromosome and the pattern of X inactivation (Bouayed Abdelmoula et al., 2003; Gunes et al., 2013). It has been reported that when a patient shows a normal male phenotype that is most probably attributed to a larger *SRY*-positive fragment being translocated to the X chromosome, during which the *SRY* gene may be protected from silencing by the spread of XCI and thus accounts for the gonadal ambiguity (Wu et al., 2014). It is possible that the *SRY*-positive fragment identified in this case might also have been translocated to the X chromosome and gave rise to a possibility of skewed inactivation during which the *SRY* gene exempted from inactivation and fully represented, which thus drove the male sexuality differentiation, but we failed to verify the *SRY*-positive fragment loci by FISH due to lack of sufficient samples. Even so, it is quite likely that the proband would show azoospermia or oligozoospermia in his puberty and ensuing infertility due to deletions in several *AZF* subregions. Because of the literature-supported risk of DMD caused by the identical p.Arg3034Ter mutation and the *SRY*-positive fragment detected in both the cord blood and labor-induced tissue samples, the mother decided to have an abortion. However, what needs to be emphasized is that the probability of development of DMD manifestations and the final phenotype of the fetus would depend on the pattern of X inactivation.

In conclusion, we evaluated the clinical manifestations and molecular features of a fetus proband who was the first 46,XX testicular DSD case diagnosed during pregnancy and carrying a mutation in dystrophin (*DMD*) gene simultaneously by integrated analysis. This research emphasized that integrating the imaging results, cytogenetics, and molecular features could play an important role in prenatal diagnosis, especially in cases when imaging examination and cytogenetic results are inconsistent. Additionally, NGS could facilitate genetic diagnosis, being a supplement of traditional clinical detection methods, which could be used in routine clinical applications. This study also suggested that genetic counseling/analysis for early diagnosis, pre-symptom interventions and disease management were warranted. In this case, we made a diagnosis of 46,XX testicular DSD by ultrasound for morphological abnormality, cytogenetics, and NGS for molecular feature details. Then, genetic counselors explained the genetic testing results and the pros and cons of each optional strategy to the proband’s mother and she made a decision of induced abortion. However, there are still many ethical issues during the counselling processes. Genetic counselors should always keep in mind whether the information they provide is comprehensive and whether the patient would be able to make an informed decision or not. Then again, despite that pathogenic mutations and CNVs were identified in the proband, other factors such as diseases’ clinical heterogeneity, variable expressivity and environmental elements can also affect the proband’s final phenotype. Genetic counseling has always had the uncomfortable shadow of eugenics looming over the field and it is important to note that abortion was not the only option.

## Data Availability Statement

The data sets used and/or analyzed during the current study are available from the corresponding authors on reasonable request. The data reported in this study are available in the CNGB Nucleotide Sequence Archive (CNSA: https://db.cngb.org/cnsa; accession number CNP0000623).

## Ethics Statement

The studies involving human participants were reviewed and approved by both the IRBs of BGI and Chenzhou No.1 People’s Hospital. Written informed consent to participate in this study was provided by the participants’ legal guardian/next of kin.

## Author Contributions

JD conducted the NGS procedure and subsequent analysis/interpretation. HH and JW conducted the genetic counseling process. SL and KX contributed to the follow up. HZ and CL performed the G-Banding cytogenetical analysis and multiple PCR for AZF deletion analysis. QW conducted the cellular histopathological analysis. DL and HY instructed and supervised this study. The manuscript was drafted by JD and edited by JW. All authors have read and approved the manuscript.

## Funding

This research was supported by the funding of the Ministry of Science and Technology of Chenzhou (No. CZ2014012 and No.CZKJ2016037).

## Conflict of Interest

The authors declare that the research was conducted in the absence of any commercial or financial relationships that could be construed as a potential conflict of interest.

## Footnotes

1 https://webpath.med.utah.edu/HISTHTML/HISTOTCH/HISTOTCH.html.

2 http://dgv.tcag.ca/dgv/app/home8:33 PM 1/7/2020

## Abbreviations

ACMG, American College of Medical Genetics and Genomics; AZF, azoospermia factor; array CGH, array comparative genomic hybridization; CNV, copy number variation; DGV, Database of Genomic Variants; DMD, Duchenne muscular dystrophy; FISH, fluorescence *in situ* hybridization; NGS, next-generation sequencing; PCR, polymerase chain reaction; SRY, sex-determining region Y; STSs, sequence-tagged sites; TDF, testis determining factor; VUS, variant of unknown significance; WGS, whole genome sequencing.
